# Travel abroad increases sexual health risk-taking among Swedish youth: a population-based study using a case-crossover strategy

**DOI:** 10.1080/16549716.2017.1330511

**Published:** 2017-06-09

**Authors:** Mats Sundbeck, Anette Agardh, Per-Olof Östergren

**Affiliations:** ^a^ Social Medicine and Global Health, Lund University, Malmö, Sweden

**Keywords:** Sexual, risk-taking, youth, casual partner, condom, travel, case-crossover

## Abstract

**Background**: The fact that youth take sexual risks when they are abroad have been shown in previous studies. However, it is not known if they increased their sexual risk-taking when travelling abroad, compared to the stay in their homeland.

**Objective**: To assess whether Swedish youth increased their individual sexual risk behaviour, defined as having a casual sex partner, when travelling abroad and to examine possible factors that may be associated with increased risk-taking abroad.

**Design**: In 2013, a population-based sample of 2189 Swedes, 18-29 years, was assessed by a questionnaire (45% response rate). Sexuality, duration of travel, parents’ country of origin, mental health, heavy episodic drinking (HED), use of illicit drugs, and socio-demographic background were assessed. Increased risk of casual sex in relation to time spent abroad vs. time spent in Sweden was analysed by a variant of case-crossover design. Factors that could be associated with increased risk of casual sex in Sweden and abroad, separately, were analysed by logistic regression.

## Background

Several studies have shown that travellers take sexual health risks when going abroad [1], which entails an increased risk of acquiring sexually transmitted infections (STIs) [2–5]. In the Swedish population, the incidence of chlamydia infections acquired abroad increased by 46% between 2006 and 2015. The incidence of gonorrhoea acquired abroad, in the population, increased by 110% between 2006 and 2015. A majority of those infected were youth between 15 and 29 years of age. Of the prevalent HIV cases, 11% were infected abroad [6,7].

About 70% of Swedish youth 15–29 years of age reported in 2008 that they were abroad at least once during the previous year [8]. Considering the proportion of STIs acquired abroad, this makes youth a particularly relevant group for targeted interventions against increased sexual health risks while abroad.

Having a casual partner [9–14], or multiple partners [9–17], or not using or inconsistently using a condom [9–11,13,14,16,17] are commonly used indicators of risky sexual behaviours. Behaviours such as indulging in heavy episodic drinking (HED), using illicit drugs, making long-time journeys, having same-sex relations, and having characteristics such as male sex, single marital status, or belonging to the age group ‘youth’ (defined by the World Health Organization (WHO) as 15–24 years of age [18]) have all been associated with sexual risk-taking abroad [9,10,12–14,16,17,19]. The majority of these studies have used convenience sampling [10,13,16,17,19], performed at e.g. a STI-clinic [17], back-packer hostels [10,13], or popular destinations for youth [16,19].

In a previous study, we found, only among men, that parents born abroad were associated with having a casual partner abroad and that poor mental health was associated with non-use of condoms with casual partner abroad [14].

Although several studies have shown similar patterns of sexual risk-taking at home and during a trip abroad [9–11,15,16], only one of them [13] compared the outcomes in relation to the length of time spent at home and abroad, respectively. Thus, empirical knowledge about whether youth in general *change* their individual sexual risk-taking when travelling abroad and whether the increase of acquired infection is mostly a spin-off effect of travel duration is very sparse. Improving this knowledge is essential for designing appropriate interventions for individuals travelling abroad. If the increasingly high number of young individuals who acquire STIs abroad is due to behavioural change or a higher risk of infection due to contextual factors (e.g. higher prevalence of the infections in question) and not only a reflection of the fact that they spend more time abroad, this would require different intervention strategies than those already applied for the domestic scene.

Being abroad could be a proxy for many circumstances that could affect sexual risk-taking, such as availability of new partners and/or contextual circumstances that facilitate or restrain sexual contact or that interact with individual determinants of sexual behaviour (e.g. gender attitudes, attitudes regarding same-sex relations). These factors could theoretically either increase or decrease sexual risk-taking. Moreover, the effect could be very different depending on age, gender, sexual orientation, type of travel abroad, and the cultural and socioeconomic context in the travel destination. However, a first step towards gaining more knowledge regarding sexual risk-taking when travelling abroad would be to confirm or refute the assumption that individuals actually change their sexual risk-taking when abroad and to investigate whether such a change could be related to basic characteristics such as gender, age, and type of travel. Therefore, studies are needed that take time spent abroad into consideration, when analysing whether being abroad is associated with a higher level of sexual risk-taking together with the same set of determinants as when at home.

The case-crossover design, first described by Maclure in 1991, was developed to study whether certain exposures of comparatively short duration increased the risk of acute onset of disease, e.g. whether sexual intercourse increased the risk of having an acute myocardial infarction [20]. In this design, each individual serves as his/her own control, in that the risk of having onset of the outcome during time units spent when not exposed to the risk factor is compared to the risk of having the same outcome during time units spent when actually being exposed to the particular risk factor. For a study population, the number of events when being exposed or being unexposed, respectively, are divided by the pooled time during which the included individuals were exposed to the risk factor or not, respectively [21]. The basic principle of comparing the risk for a certain event for one particular individual in one context (e.g. being in one’s native country) to the risk for the same type of event in another context (e.g. being abroad) can therefore justify the use of a case-crossover design for studying change in sexual risk-taking when travelling abroad.

The aim of this study was to assess whether Swedish youth increase their sexual risk-taking behaviour when travelling abroad, in terms of having sex with a casual partner, and to examine possible factors that may be associated with such increased risk-taking abroad.

## Methods

### Study setting

The study was performed in Skåne, the southernmost county in Sweden with 1.3 million inhabitants, among whom approximately 206,000 were 18–29 years of age in 2013. Southern Sweden borders on Denmark, and an interconnecting bridge provides easy access to entertainment on both sides of the border and also increased employment opportunities within commuting distance for Swedish youth.

### Participants, instrument and data collection

The potential participants were randomly drawn from the Swedish Central Population Registry and consisted of 7000 individuals between the ages of 18 and 29 years with permanent residence in Skåne on 1 January 2013.

The selected individuals were asked to respond to a 79-item self-report questionnaire, which focused on sexuality, lifestyle, and health. The questionnaire contained pre-validated questions about socio-demographic factors, social capital, general health, mental health, experiences of sexual coercion, use of drugs and alcohol, and sensation-seeking behaviour.

Data collection took place between January and March 2013. All invited participants received an introductory letter with information about the study, the voluntary nature of participation, and a guarantee of anonymity. The letter also included a link to the online questionnaire. Three reminders were sent out, and the last one included a printed version of the questionnaire. After completing the questionnaire, the respondents received a cinema ticket as compensation.

Since the study aimed to examine the possible increased sexual risks when travelling abroad, we excluded all respondents who reported that they had not been abroad (n = 504) or those who had not been sexually active (defined as vaginal or anal intercourse or oral sex) during the last 12 months (n = 331), and those who did not state their gender (n = 23). These groups overlapped to some extent, and all in all, 779 persons were excluded and consequently 2189 remained for further analysis.

### Independent variables

*Gender* was classified as men or women.

*Age* was dichotomised as 18–24 years and 25–29 years of age, motivated by previous findings that younger individuals take more sexual health risks [9,12,16].

*Level of parents’ education* was used as an indicator of socioeconomic status since younger persons might not have finished their education or might lack regular employment or income. Parental education can be used as a predictor of youth occupational outcome [22]. The response alternatives were: ‘9-year compulsory school’, ‘2-years of upper secondary school’, ‘3–4-years of upper secondary school’, ‘other types of schools’, and ‘university’. This variable was then dichotomised as ‘University’ if a parent had a university degree, and ‘Less than university’ for all other alternatives.

*Parents’ country of origin* was used to identify youth with an immigrant background, as experience of and attitude to sexuality can vary with cultural background [14]. The alternatives were: ‘Both parents born in Sweden’, ‘One of the parents born abroad’, or ‘Both parents born abroad’. The answers were dichotomised as ‘Both parents born in Sweden’ or ‘At least one parent born abroad’.

#### Self-rated mental health

Poor mental health has previously been shown to be associated with sexual risk-taking [23]. Mental health status was measured using the Hopkins Symptom Checklist (HSCL-25) [24], a self-reporting instrument that assesses symptoms of anxiety (10 items) and depression (15 items) on a scale from 1 (‘not at all’) to 4 (‘extremely’). The HSCL-25 has been used and validated in different cultural settings including Sweden [25]. For each item, respondents were asked, ‘How much has this problem bothered or distressed you during the last month, counting today?’. Mean total mental health scores, as well as mean scores for depression and anxiety, were calculated on the basis of a respondent’s total scores. The scores were summed up and divided by the number of answered items to generate a symptom mean score ranging from 1 to 4. The mean scores were dichotomised as ‘satisfactory self-rated mental health’, i.e. ‘low HSCL symptom score’, and ‘poor self-rated mental health’, i.e. ‘high HSCL symptom score’, based on the median split of the frequency distributions of the respondents’ individual mean mental health scores. Due to a technical error, the item ‘thoughts of ending one’s life’ was not included in the paper version of the questionnaire. Consequently, about one-fifth of the respondents did not have access to this question and the question was excluded from all analyses.

#### Travelling abroad

The question was: ‘Have you been abroad during the last 12 months?’. The alternatives were ‘Yes, not counting Denmark’, ‘Yes, only in Denmark’, and ‘No’. Responses were dichotomised as ‘Yes’ if the respondent had been in any foreign country, including Denmark, and ‘No’ if the respondent had not travelled abroad at all during the previous year.

#### Duration of most recent travel

The question was ‘How long a time did you spend abroad during your last stay abroad during the last 12 months?’ We considered this representative of travels abroad for each individual, since asking for the most recent travel is a random selection mechanism. Response alternatives for the stay abroad were: 1 day, 2–6 days, 7–8 days, 9–29 days, 1–6 months, and more than 6 months. The six travel time intervals were reduced and categorised as four intervals using the median day for each response category: 5 days, 19 days, 105 days, and 270 days, respectively, as an approximation of the actual time spent abroad. A dichotomous outcome was also constructed for this variable; individuals who had spent = 29 days abroad were classified as short-time travellers and those with longer stay as long-time travellers.

*Time spent in Sweden* was calculated as 365 days minus the approximation of the actual time spent abroad (see earlier) for a particular individual, i.e. resulting in 360 days, 346 days, 260 days, or 95 days. Since we only collected information regarding the most recent travel abroad, we lacked information about the accumulated time spent abroad during the last year. However, since long stays were rather unusual in the study sample, we considered this to be a minor potential source of bias.

*Heavy episodic drinking* (HED) was defined by the question, ‘How often did you consume at least five (if you are a man) or four (if you are a woman) “glasses” at the same occasion during the last 12 months?’ One unit is 1.5 fl. oz. or 4.4 cl of 40% alcohol or an equivalent amount of alcohol in beer or wine [26]. The response alternatives were ‘every day or nearly every day’, ‘every week’, ‘2–4 times/month’, ‘every month’, and ‘less than once a month’, or ‘never’. Individuals who reported high consumption (the three first mentioned frequencies) were categorised as ‘HED’ and the others were categorised as ‘non-HED’. The question was posed separately regarding while in Sweden or while travelling abroad. HED has been shown to be associated with sexual risk-taking abroad [16].

#### Use of illicit drugs

This item was based on the question, ‘Have you during the last 12 months used illicit drugs?’ Examples were given such as cannabis, cocaine, and amphetamine. Illicit drug use has been shown to be associated with increased sexual risk-taking [16]. The response alternatives were ‘Yes’ or ‘No’. The question was posed separately regarding while in Sweden or while travelling abroad.

### Dependent variables

#### The relation to the last sexual partner

The question was, ‘What relation do/did you have to the last person you had sexual intercourse with?’ The response alternatives were dichotomised by classifying the response alternatives ‘married, living together or in a steady relationship’ as ‘regular partner’ and ‘previous partner’, ‘friend’, ‘casual contact’, ‘commercial sex partner’, or ‘another non-regular partner’ as ‘casual partner’. The question was posed separately regarding while in Sweden or while travelling abroad.

### Statistical analysis

As a first step, the relationships between the outcomes ‘casual partner in Sweden’ and ‘casual partner abroad’ and the independent factors were examined by binary regression analyses and chi-square tests. Associations were examined, for both genders separately, in relation to the potential confounders represented by age, parents’ country of origin, duration of most recent travel, HED, use of illicit drugs, and mental health when simultaneously adjusted for one another, with results presented in terms of odds ratios (OR) and 95% confidence intervals (CI).

A case-crossover design was then applied. The last time abroad during the last 12 months constituted the ‘exposure window’, and time spent in Sweden during the same year was set as the ‘reference window’. The outcome (‘cases’) in both windows was an occasion of having a casual partner. The time spent in the exposure window (abroad) and reference window (Sweden), respectively, was pooled as the person-time constituting the denominator for calculating the incidence of risk behaviour in the respective context. The risk was then determined as the ratio between those incidences, i.e. as an Incidence Rate Ratio (IRR). Furthermore, the travellers were split into four ‘travel-time groups’ according to the time they spent abroad, and IRRs were calculated for all travel-time groups, with separate analyses for women, men, and for the entire sample. Calculations were made using OpenEpi version 3.03a [27] and SPSS version 22. Statistical significance was accepted at *p* = 0.05.

## Results

Of the 7000 letters of invitation sent, 332 letters came back by return post, mostly due to incorrect address information. A total of 2968 persons responded to the questionnaire, representing 45% of the entire number of recipients. Of the respondents, 82% answered electronically and 18% by mail. As mentioned earlier, only individuals who reported that they had travelled abroad and who had been sexually active were retained in the analyses, which rendered a final sample of 2189 individuals.

Table 1 shows the distribution of socio-demographic and background characteristics of the sample. The majority were women (59%), 18–24 years of age (58%), and had spent = 29 days (short-time travellers) abroad (90%).

**Table 1.** Distribution of socio-demographic and background characteristics, including lifestyle and sexual risk-taking, among youth in Sweden who had travelled abroad during the last 12 months (n = 2189).

	Sweden = 12 months											
	All		Men		Women							
	n	%	n	%	n	%						
*Gender*												
Number	2189		897	41	1292	59						
*Age*												
18–24 years of age	1260	58	509	57	751	59						
25–29 years of age	913	42	381	43	532	41						
(Missing)	(16)		(7)		(9)							
*Level of parents’ education*												
University	1361	63	547	61	814	63						
Less than university	814	37	344	39	470	37						
(Missing)	(14)		(6)		(8)							
*Parents’ country of origin*												
Both parents born in Sweden	1620	74	650	73	970	75						
At least one parent												
born abroad	560	26	241	27	319	25						
(Missing)	(9)		(6)		(3)							
*Self-rated mental health*												
Satisfactory mental health	1162	53	597	67	565	44						
Poor mental health	1023	47	297	33	726	56						
(Missing)	(4)		(3)		(1)							
							Last trip abroad = 12 months					
							All		Male		Female	
							n	%	n	%	n	%
*Duration of most recent travel*												
1–8 days							1448	67	581	66	867	68
9–29 days							488	23	209	24	279	22
1–6 months							167	8	67	8	100	8
= 6 months							53	2	18	2	35	2
(Missing)							(33)		(22)		(11)	
*Relation to the last sexual partner*												
Regular partner	1517	70	549	62	968	76	678	75	245	69	433	80
Casual partner	639	30	332	38	307	24	222	25	112	31	110	20
(Missing)	(33)		(16)		(17)		(0)		(0)		(0)	
*Heavy episodic drinking (HED)*												
Non-HED	932	46	306	36	626	52	926	46	317	38	609	51
HED	1115	54	541	64	574	48	1100	54	518	62	582	49
(Missing)	(142)		(50)		(92)		(163)		(62)		(101)	
*Use of illicit drugs*												
No	1918	88	737	83	1181	92	2016	94	791	90	1225	96
Yes	263	12	154	17	109	8	139	6	87	10	52	4
(Missing)	(8)		(6)		(2)		(34)		(19)		(15)	

Thirty-three percent of the men and 56% of the women rated their mental health as poor. HED when travelling abroad was more common among men compared to women (62% and 51%, respectively). Men were twice as likely to use illicit drugs compared to women, both in Sweden (17% and 8%, respectively) and abroad (10% and 4%, respectively).

One out of four individuals who had sex during their last stay abroad (n = 900) reported that their last sexual partner abroad was casual (n = 222). Nearly 30% (n = 639) of the total sample (n = 2189) reported that their last partner in Sweden was casual. Casual partners were more common among men than among women, both when in Sweden and when travelling abroad.

First, we performed logistic regression to examine factors associated with having a casual partner in Sweden and when abroad. Having a casual partner abroad was more common in men compared to women (OR 1.80, CI 1.33–2.44) (not shown in tables). Therefore, the analyses were split by gender to investigate whether the behavioural determinants differed between the two genders.

Tables 2 and 3 show that the following variables were significantly associated with having a casual partner in Sweden, among both men and women: younger age (18–24 years), HED, and illicit use of drugs. However, poor mental health was associated with casual sex in Sweden solely among men. The same factors were also associated with having a casual partner abroad, except that poor mental health was no longer significantly associated with having a casual partner among men. Moreover, long-time travellers (men and women) were significantly more likely to have casual partners when abroad, when travel duration was dichotomised.

**Table 2.** Men (n = 897). Crude and adjusted* associations (odds ratio [OR], 95% confidence interval [CI]) for men whose last sex partner in *Sweden* during the past year was casual (n = 332) and men whose last sex partner *abroad* during the past year was casual (n = 112) in relation to age, parents’ country of origin, duration of travel, heavy episodic drinking (HED), use of illicit drugs, and self-rated mental health.

	Men who have had casual sex partner in Sweden						Men who have had casual sex partner abroad					
	Crude			Adjusted			Crude			Adjusted		
	OR	CI	(m)	OR	CI	(m)	OR	CI	(m)	OR	CI	(m)
*Age*			(23)			(79)			(5)			(29)
25–29 years	Ref. 1			Ref. 1			Ref. 1			Ref. 1		
18–24 years	**2.42**	**1.81–3.23**		**2.45**	**1.8–3.33**		**2.08**	**1.31–3.30**		**2.40**	**1.43–4.02**	
*Parents’ country of origin*			(22)						(3)			
Both parents born												
in Sweden	Ref. 1			Ref. 1			Ref. 1			Ref. 1		
At least one parent born abroad	1.26	0.93–1.71		1.36	0.96–1.93		**1.89**	**1.16–3.08**		1.49	0.83–2.67	
*Duration of most recent travel*									(5)			
Long-time travel							**2.69**	**1.51–4.80**		**2.32**	**1.18–4.56**	
Short-time travel							Ref. 1			Ref. 1		
*Heavy episodic drinking*			(65)						(17)			
Non-HED	Ref. 1			Ref. 1			Ref. 1			Ref. 1		
HED	**2.13**	**1.56–2.90**		**1.96**	**1.42–2.72**		**2.52**	**1.46–4.35**		**2.23**	**1.25–3.98**	
*Use of illicit drugs*			(21)						(3)			
No	Ref. 1			Ref. 1			Ref. 1			Ref. 1		
Yes	**2.39**	**1.67–3.40**		**1.92**	**1.3–2.83**		**3.71**	**1.87–7.35**		**2.38**	**1.10–5.16**	
*Self-rated mental health*			(19)						(1)			
Satisfactory mental health	Ref. 1			Ref. 1			Ref. 1			Ref. 1		
Poor mental health	**1.59**	**1.15–2.12**		**1.49**	**1.09–2.05**		1.30	0.81–2.1		1.41	0.81–2.44	

Notes: *All variables are simultaneously adjusted for one another.

(m) = missing.

**Table 3.** Women (n = 1292). Crude and adjusted* associations (odds ratio [OR], 95% confidence interval [CI]) for women whose last sex partner in *Sweden* during the past year was casual (n = 307) and women whose last sex partner *abroad* during the past year was casual (n = 110) in relation to age, parents’ country of origin, duration of travel, heavy episodic drinking (HED), illicit use of drugs, and self-rated mental health.

	Women who have had casual sex partner in Sweden						Women who have had casual sex partner abroad					
	OR	CI	(m)	OR	CI	(m)	OR	CI	(m)	OR	CI	(m)
	Crude			Adjusted			Crude			Adjusted		
*Age*			(26)			(117)			(6)			(55)
25–29 years	Ref. 1			Ref. 1			Ref. 1			Ref. 1		
18–24 years	**2.15**	**1.63–2.84**		**2.42**	**1.81–2.59**		**3.14**	**1.95–5.05**		**2.99**	**1.75–5.10**	
*Parents’ country of origin*			(20)						(1)			
Both parents born in Sweden	Ref. 1			Ref. 1			Ref. 1			Ref. 1		
At least one parent born abroad	0.83	0.61–1.12		0.87	0.62–1.22		0.78	0.47–1.29		1.04	0.58–1.87	
*Duration of most recent travel*									(2)			
Long-time travel							**2.73**	**1.64–4.55**		**1.87**	**1.01–3.45**	
Short-time travel							Ref. 1			Ref. 1		
*Heavy episodic drinking*			(106)						(44)			
Non-HED	Ref. 1			Ref. 1			Ref. 1			Ref. 1		
HED	**3.17**	**2.40–4.19**		**2.71**	**2.03–3.62**		**3.78**	**2.27–6.30**		**3.31**	**1.91–5.72**	
*Use of illicit drugs*			(19)						(4)			
No	Ref. 1			Ref. 1			Ref. 1			Ref. 1		
Yes	**3.19**	**2.14–4.78**		**2.11**	**1.37–3.25**		**5.19**	**2.55–10.5**		**4.81**	**2.14–10.8**	
*Self-rated mental health*			(18)						(1)			
Satisfactory mental health	Ref. 1			Ref. 1			Ref. 1			Ref. 1		
Poor mental health	**1.33**	**1.02–1.73**		1.15	0.88–1.53		1.38	0.9–2.12		1.19	0.73–1.94	

Notes: *All variables are simultaneously adjusted for one another.

(m) = missing.

We then applied the modified case-crossover design to further explore the risk of having a casual partner abroad, using more detailed information concerning duration of travel.

Table 4 shows the relative risk expressed as IRRs in the case-crossover analyses, of sex with a casual partner in four different ‘travel-time groups’ by the median days spent in Sweden and during the latest trip abroad. Overall, the risk of having a casual partner while abroad compared to while in Sweden was fivefold increased (IRR 5.37, CI 4.61–6.26). Men and women had a similar level of increased risk for having a casual partner when abroad. All groups that spent the majority of their days in Sweden had an increased risk of sex with a casual partner abroad. In contrast, those with a median of 270 days abroad and 95 days in Sweden had a decreased risk of sex with a casual partner abroad (IRR 0.3, CI 0.15–0.58).

**Table 4.** Incidence Rate Ratios (IRR) of casual sex partner in a sample of Swedish youth (n = 2189, missing n = 33) who reported that they had travelled abroad during the past year. A modified case-crossover analysis of events of casual sex in Sweden and abroad in relation to median days spent in Sweden and median days in last travel abroad, during the last 12 months (95% confidence interval [CI], two-tailed *p*-value).

		Median days in Sweden/abroad^1^		Individuals who have had a casual sex partner						
	Total number of individuals	Sweden	Abroad	Sweden	(m)^2^	Abroad	(m)	IRR	(CI)	*p*
Total	2156	738,583	48,357	626	(13)	221	(1)	5.37	(4.61–6.26)	< 0.001
Men	875	300,604	18,771	320	(12)	111	(1)	5.53	(4.46–6.86)	< 0.001
Women	1281	437,979	29,586	306	(1)	110		5.31	(4.27–6.6)	< 0.001
Data regarding number of days spent in Sweden and abroad	1448	360	5	412		110		18.9	(15.4–23.4)	< 0.001
	488	346	19	126		52		7.45	(5.41–10.3)	< 0.001
	167	260	105	69		43		1.54	(1.05–2.26)	< 0.036
	53	95	270	19		16		0.3	(0.15–0.58)	< 0.001

Notes: ^1^1–8 days (median time 5 days), 9–29 days (median time 19 days), 1–6 months (median time 105 days), and more than 6 months (median time 270 days). The median days spent in Sweden are consequently: 360 days, 346 days, 260 days, and 95 days.

^2^(m) = Missing.

## Discussion

The main findings in this study are that the risk of having sex with a casual partner while abroad increased about fivefold for both genders in a general population sample of youths in southern Sweden. However, the time spent abroad had a strong modifying effect on the risk of having a casual partner. Thus, those who had spent a very short time (around 5 days) abroad had an almost 20-fold increased risk of having had sex with a casual partner abroad per time unit.

The main focus of this study was to test the hypothesis that travelling abroad increases the tendency to engage in riskier sexual behaviour. Previous studies have supported this notion, and it seems that an increasing proportion of the most common forms of STIs diagnosed among Swedish youth is acquired abroad. However, previous studies have not been designed to determine whether individuals who already practised risky sexual behaviour in their usual environment continued to do so while abroad and thus acquired STIs more often because of their higher prevalence in foreign sexual partners than in sexual partners in Sweden, or alternatively, whether being abroad changed the individual’s behaviour in a riskier direction, e.g. because of less social control or a higher presence of triggering factors.

Therefore, this study applied a modified case-crossover study design. This approach was developed for studying risk factors for acute disease that are of short duration, e.g. to see if anger triggers myocardial infarction [21]. The general idea is that the individual serves as his/her own control, in that the occurrence of the acute event is allocated to the time spent in the risky behaviour or time spent when not in the risky behaviour. Since we lacked information about the actual number of events in each exposure window, the single recorded event in the two windows should be regarded as a measure of the probability of the risky behaviour (i.e. having a casual sex partner vs. a regular one on the most recent occasion of sexual intercourse). However, taking person-time into consideration is still very relevant since the majority of the individuals did not report a risk event in either of the two contexts (Sweden vs. abroad).

The advantage of the case-crossover design rests in the fact that each individual contributes with risk time for both exposure and non-exposure to the risky behaviour. In practice this means that there is no need for adjustment for any potential confounders in the analysis, since the same individuals are used for assessing risk time for both the exposed and the unexposed group. Thus, in theory, the increased risk depends solely on the studied exposure (here: being abroad). This study design therefore has the potential to separate the two different possible causal mechanisms behind the increased incidence of STIs among young Swedes who have been abroad, i.e. change in behaviour while abroad or change in risk of getting infected because of environmental factors (i.e. no significant behaviour change).

Our findings provide strong support for the first of those two hypotheses, i.e. being abroad actually seems to have a very strong impact on a particular individual’s propensity to engage in risky sexual behaviour. Previous studies [10,28] have also shown that youth going abroad frequently have an expectation of having casual sex while abroad. The extent to which the amount of time spent abroad modified the effect of travel could be interpreted in several ways. One reasonable interpretation could be that length of stay may be a proxy for different types of trips abroad. For example, it cannot be discounted that some of the shorter trips in fact are made with the intention to engage in sex with a new partner.

The fact that the individuals in our study with the longest time spent abroad actually had lower odds of having a casual partner compared with being in Sweden could imply very different reasons for being abroad, e.g. work, or a family situation that might serve as barriers for engaging in casual sex. The bottom line of all these scenarios is that being abroad indeed affects the individual’s behaviour, although in different ways depending on the purpose of the travel/stay abroad. The results suggest that interventions aiming at reducing the increasing proportion of STIs among young Swedes acquired abroad should not primarily focus on factors that affect risky behaviour while in Sweden. Furthermore, when designing such interventions, the departure point should be that being abroad seems to change youths’ behaviour depending on the specific type of travel/stay abroad, and in most cases, such that risky sexual behaviour increases. Supplementary qualitative studies could shed more light on the specific mechanisms behind our findings.

Our findings that lower age, HED, and use of illicit drugs, in both men and women, could be associated with sexual risk-taking both at home and abroad, are consistent with findings in previous studies [9,11–13,16,17]. Both HED and illicit use of drugs may increase while travelling abroad and thus interact in our major findings – so that the trip itself has the effect of increasing the risk. But both at home and abroad, HED as well as use of illicit drugs are significantly more common among those who have a casual partner. This applies to both men and women (see Tables 2 and 3). In order to adequately assess the extent to which individuals may increase their consumption of alcohol and illicit drugs while travelling, a different and a much more detailed study of consumption habits is needed both in Sweden and abroad. Future studies should take this into account.

### Methodological considerations

The information used in this study was obtained by retrospective self-reporting. The possibility of recall bias or non-dependent under- or over-reporting cannot be entirely excluded. This could lead to overestimations of the shown associations as well as to underestimations. The relatively low response rate (45%) could give rise to a selection bias, which also could bias our findings in any direction. In the present study, men and youth 18–24 years of age were underrepresented and persons having parents with higher education were overrepresented. An underrepresentation of youth aged 18–24 years can presumably give rise to an underestimation of sexual risk-taking abroad. These findings are consistent with participation patterns in epidemiological studies [29]. Remuneration to increase the participation rate can lead to selection bias. Nevertheless, a cinema ticket presumably has such low value today for most young people in Sweden that no particular group would feel more tempted to participate than other groups.

Another possible limitation is that the results solely concern the last intercourse in Sweden and last time abroad. Thus, the duration of the last trip is used to represent the time spent abroad during the previous year. This choice was prompted by the need to reduce recall bias regarding sexual risk-taking. We argue that, in a population perspective, the last travel abroad and the last occasion of sexual intercourse were reasonably representative of all occasions. However, it will cause a systematic misclassification of the estimated time spent abroad, i.e. an underestimation of this time. This bias is proportionate to the duration of the reported most recent travel abroad. Therefore, those risk estimates are likely to be somewhat inflated which warrants some caution regarding the steepness of the found gradient. However, we judge it very improbable that the gradient could be explained solely by this factor. Another limitation is that information was lacking concerning the travel purpose, which prevents any closer examination of why shorter trips (five days) were associated with the very greatest risks.

The case-crossover method, using the individual as both case and control, reduces confounders as the individual characteristics are constant throughout the observation period. The modification of this study design, i.e. including all individuals who had spent time abroad to contribute in order not to reduce the sample and thus reduce statistical power to a critical extent, could have re-introduced some confounding in our results, but sensitivity analysis indicated that this probably was a minor source of bias.

The strengths of the study are its size, that it is one of the few that has focused on youth in a general population sample, and that it is the first of its kind in Scandinavia. The results are deemed generalisable to youth in other regions in Sweden and in similar countries who are sexually active during travel abroad.

## Conclusion and future recommendations

Both women and men showed an increased risk of having casual sex while travelling abroad compared to while staying in Sweden. Future research should examine the underlying reasons that contribute to this increased risk, in order to be able to design appropriate interventions for young travellers. Both the increasing popularity of travel abroad among today’s youth and the high level of sexual activity in this age group in general indicate that interventions are needed to address this growing public health challenge.

## Paper context

That youth take sexual risks when going abroad is shown in several studies. However, it is not known whether youth are increasing or decreasing their risk-taking when they go abroad. By using a variant of a case-crossover method, and taking time into consideration, we show that Swedish youth increase their sexual risk-taking while abroad and that the travel abroad in itself increases sexual risk-taking. This knowledge is important in the design of intervention strategies.
